# Distinct genes and pathways associated with transcriptome differences in early cardiac development between fast- and slow-growing broilers

**DOI:** 10.1371/journal.pone.0207715

**Published:** 2018-12-05

**Authors:** Jibin Zhang, Carl J. Schmidt, Susan J. Lamont

**Affiliations:** 1 Department of Animal Science, Iowa State University, Ames, IA, United States of America; 2 Department of Animal and Food Sciences, University of Delaware, Newark, DE, United States of America; Northwestern University, UNITED STATES

## Abstract

Modern fast-growing broilers are susceptible to cardiac dysfunctions because their relatively small hearts cannot adequately meet the increased need of pumping blood through a large body mass. To improve cardiac health in broilers through breeding, we need to identify the genes and pathways that contribute to imbalanced cardiac development and occurrence of heart dysfunction. Two broiler lines–Ross 708 and Illinois–were included in this study as models of modern fast-growing and heritage slow-growing broilers, respectively. The left ventricular transcriptome were compared between the two broiler lines at day 6 and 21 post hatch through RNA-seq analysis to identify genes and pathways regulating compromised cardiac development in modern broilers. Number of differentially expressed genes (DEGs, p<0.05) between the two broiler lines increased from 321 at day 6 to 819 at day 21. As the birds grew, Ross broilers showed more DEGs (n = 1879) than Illinois broilers (n = 1117). Both broilers showed significant change of muscle related genes and immune genes, but Ross broilers showed remarkable change of expression of several lipid transporter genes including *APOA4*, *APOB*, *APOH*, *FABP4* and *RBP7*. Ingenuity pathway analysis (IPA) suggested that increased cell apoptosis and inhibited cell cycle due to increased lipid accumulation, oxidative stress and endoplasmic reticulum stress may be related to the increased cardiac dysfunctions in fast-growing broilers. Cell cycle regulatory pathways like “Mitotic Roles of Polo-like Kinases” are ranked as the top changed pathways related to the cell apoptosis. These findings provide further insight into the cardiac dysfunction in modern broilers and also potential targets for improvement of their cardiac health through breeding.

## Introduction

After intensive genetic selection for decades since early 1950s, modern broilers have shown a strikingly high growth rate with reduced marketable age and higher carcass yield [1]. However, with the fast growth of breast muscle but compromised development of other organs, health issues have arisen in broilers including lameness due to skeletal disorders and bone deformity [2], sudden death due to cardiovascular failure [3], and ascites syndrome due to pulmonary hypertension [4]. Among these problems, cardiac arrhythmia is especially prevalent with an incidence of 27% in fast-growing broilers and only 1% in slow-growing broilers [5]. Cardiac insufficiency can deteriorate into ascites and sudden death syndrome. Therefore, genetic improvement is needed to optimize cardiac function while maintaining efficient growth in broilers.

To better understand the genetic basis for cardiac dysfunction in fast-growing broilers, it is informative to determine what has been changed during decades of selective breeding. Ross 708 and Illinois broilers provide excellent models for this research objective. Ross broilers were intensely selected for primary broiler traits of rapid growth, feed efficiency and muscle yield, as well as numerous fitness traits some of which are proprietary to the breeding company, while the Illinois broilers were crossbred between a lean-type broiler New Hampshire males and females carrying the Columbian feather pattern that were maintained in University of Illinois, Urbana under no selection for any traits since 1956 [6]. Previous study has shown that body weight of Ross broilers increased by 6.1 g/d from day 2 to 35 after hatching, which is 1.8 times faster than that of Illinois broilers. The hearts of Ross broilers grew at 316 mg/d, which is 1.3 times faster than that of Illinois broilers. Due to the negative allometric growth, however, the percentage of the Ross heart to body mass gradually became smaller. The average normalized Ross heart decreased from 0.75% at day 2 to 0.55% at day 35, with the major decrease occurring between day 14 and 21 post hatch. On the other hand, the normalized Illinois heart mass did not show significant change during this period, making it larger than the normalized Ross heart after day 14 [6]. In the 1957 Athens-Canadian Randombred Control (ACRBC) and the 1991 Arbor Acres (AA) broilers, smaller heart size as a percentage of body weight was also observed in the AA [7] which showed higher mortality than the ACRBC broilers due to sudden death syndrome and ascites [8]. Therefore, reduced relative heart size with diminished cardiac capacity in modern broilers may be a reason for their increased susceptibility to cardiac dysfunction.

In a previous study, we compared gene expression between the two broiler lines at 42 days post hatch, and found that multiple genes involved in cell cycle are differentially expressed between the two lines and “mitotic role of polo-like kinase” is a potential pathway regulating their differential cardiac growth [9]. However, gene expression patterns at earlier times have not been defined. Although it has been suggested that the higher percentage of mortality due to flip-overs and ascites in fast-growing broilers are observed mainly after day 21[8], the associated changes in molecular level may have been initiated at earlier age [10]. Therefore, we compared gene expression at day 6 and 21 between the two lines to investigate earlier change in transcriptomic regulation associated with susceptibility to cardiac dysfunction in modern-fast growing broilers.

## Materials and methods

### Broilers and experimental design

Eggs of Ross 708 and Illinois broilers were obtained from Mountaire Farm in Millsboro, Delaware, and University of Illinois at Urbana-Champaign, respectively. Chicks were hatched at the University of Delaware in a large colony house at standard industry stocking density for the duration of the 3-wk study. Chicks received continuous light and were allowed ad libitum access to feed and water for the duration of the experiment. A commercial starter ration (calculated values of 23.1% protein with 1.2% lysine, and 3,235 kcal apparent metabolisable energy / kg) was fed to both broiler lines during this study. No incidence of mortality or cardiac morbidity was observed in either Ross 708 or Illinois broilers during this study, but only male broilers, which were reported to be more susceptible to cardiac dysfunction than females [11], were used to eliminate gender-specific effects. At day 6 and 21 posthatch, 8 broilers in each line were euthanized by cervical dislocation, and their body and heart weights were measured and compared through two-way analysis of variance (ANOVA) and post hoc least significant difference test. Then 5 broilers from each line at each time point were randomly selected and their left ventricles were collected into liquid nitrogen and stored at -80°C for subsequent RNA isolation. All animal experiments were conducted following the Guide for the Care and Use of Laboratory Animals of the National Institutes of Health and the protocol (Permit No.: 2703-12-10) approved by the Committee on the Ethics of Animal Experiments of the University of Delaware.

### RNA isolation

Total RNA samples were isolated from the left ventricle of each broiler using the RNeasy Fibrous Tissue Mini Kit (Qiagen, Germantown, MD) following the manufacturer’s instruction. Concentration of the RNA samples was measured by Nanodrop ND-100 spectrophotometer (Thermo Fisher Scientific, Waltham, MA) and RNA Integrity Number (RIN) was assessed by Agilent 2100 Bioanalyzer (Agilent Technologies, Santa Clara, CA). All samples used in library construction had a RIN above 9.

### cDNA library construction and sequencing

A transcriptome library was constructed from each RNA sample using Illumina TruSeq RNA Library Prep Kit (Illumina, San Diego, CA) following the manufacturer’s instruction. Sequences of 50-bp single-end reads in each lane were obtained using the HiSeq 2500 Sequencing System (Illumina) at the Delaware Biotechnology Institute’s Sequencing and Genotyping Center (Newark, DE). All libraries were sequenced at a depth of ~10 million, 50 bp single-end reads per library. The sequencing data have been deposited in NCBI’s Sequence Read Archive database with accession number SRP149598 (https://www.ncbi.nlm.nih.gov/sra/SRP149598).

### RNA-seq analysis

A series of applications in the Discovery Environment of CyVerse (https://de.iplantcollaborative https://de.cyverse.org/de/), including FastQC (version 0.10.1), FASTX clipper and quality filter, TopHat2-SE with TopHat (version 2.0.9) and Bowtie (version 2.1.0), and HTSeq (version 0.6.1) were utilized for RNA-seq analysis. Quality assessment with FastQC followed by trimming and filtering with FASTX workflow ensured that all the libraries were of good quality with reads length>30bp and Phred score larger than 25 in all bases. Sequence reads in each trimmed and filtered library were then mapped to *Gallus gallus* Galgal 5.0 reference genome (assembly GCA_000002315.3) using TopHat2-SE with default parameters. The mapped reads per exon were counted using the HTSeq program with default parameters. The number of reads per gene was calculated and shown in the output file with Ensembl gene ID.

Principal component analysis (PCA) was performed using the Bioconductor package DEseq2 (version 1.10.1) in R software (version 3.1.3) based on variance-stabilized normalized read counts [12]. Differentially expressed (DE) genes between treatments and lines were obtained through analysis using edgeR (version 3.12.0), in which the trimmed mean of M-values method was used to minimize the effect of technical bias on the results [13] and a general linear model including age and line effects was fit to the data. The false discovery rate (FDR) of each gene in a pair-wise comparison was determined using the Benjamini-Hochberg method. Significant DE genes (DEGs) with FDR<0.05 were filtered in each comparison between different treatments or lines, which were then input into Ingenuity pathway analysis (IPA) software (Ingenuity Systems, Redwood City, CA) to analyze and predict difference in canonical pathways, and occurrence of disease and bio-functions.

### Fluidigm Biomark assay

To validate RNA-seq results, we selected 43 genes covering the full range of log2 fold change based on RNA-seq, and 3 housekeeping genes [Glyceraldehyde-3-phosphate Dehydrogenase (*GAPDH*), Hexose-6-phosphate Dehydrogenase (*H6PD*) and Ribosomal Protein S13 (*RPS13*)], and then conducted Biomark q-PCR assay (Fluidigm, South San Francisco, CA) with the same 20 RNA samples. Primers designed for each gene are shown in S1 Table. Among these, primer pairs for 29 genes are from previous studies [6,14,15], and the other 17 primer pairs were designed by Fluidigm to yield ~100bp amplicons spanning two adjacent exons. The geometric means of Ct values of the 3 housekeeping genes were used for normalization. For each sample, 50 ng of RNA was used for cDNA preparation with Reverse Transcription Master Mix (Fluidigm, South San Francisco, CA) according to the manufacturer’s protocol. To determine the proper preamplification cycle number, the 17 new plus 7 randomly selected old primer sets were tested in two partitions on a Flex-Six integrated fluidic circuit (IFC, Fluidigm) with cDNA pools of four groups separated by age and line. Each cDNA pool was preamplified for 10, 12 and 14 cycles; 14 cycles was found to be the best preamplification cycle number that allows proper Ct value for most genes. Then Biomark qPCR assay was performed with a 48.48 Dynamic Array IFC chip (Fluidigm) for individual samples in duplicates or quadruplicates at 14 cycles in BioMark HD (Fluidigm) Real-Time PCR system, and data was analyzed using Fluidigm Real-Time PCR Analysis software. Four genes [bone morphogenetic protein 10 (*BMP10*), myosin binding protein H (*MYBPH*), troponin I type 2 (*TNNI2*), myosin heavy chain 1 (*MYH1E*)] were excluded from analysis due to technical issues (frequent detection failure or nonspecific amplification as shown by their melting curve). Expression of the remaining 39 genes were compared between different groups using 2(-ΔΔCt) method and pairwise correlation and linear regression analysis was performed between Log2FC in RNA-seq and -ΔΔCt in Biomark assay in JMP 13 software.

## Results

### Change of body and heart weights of two broiler lines from day 6 to 21

**Fig 1:**
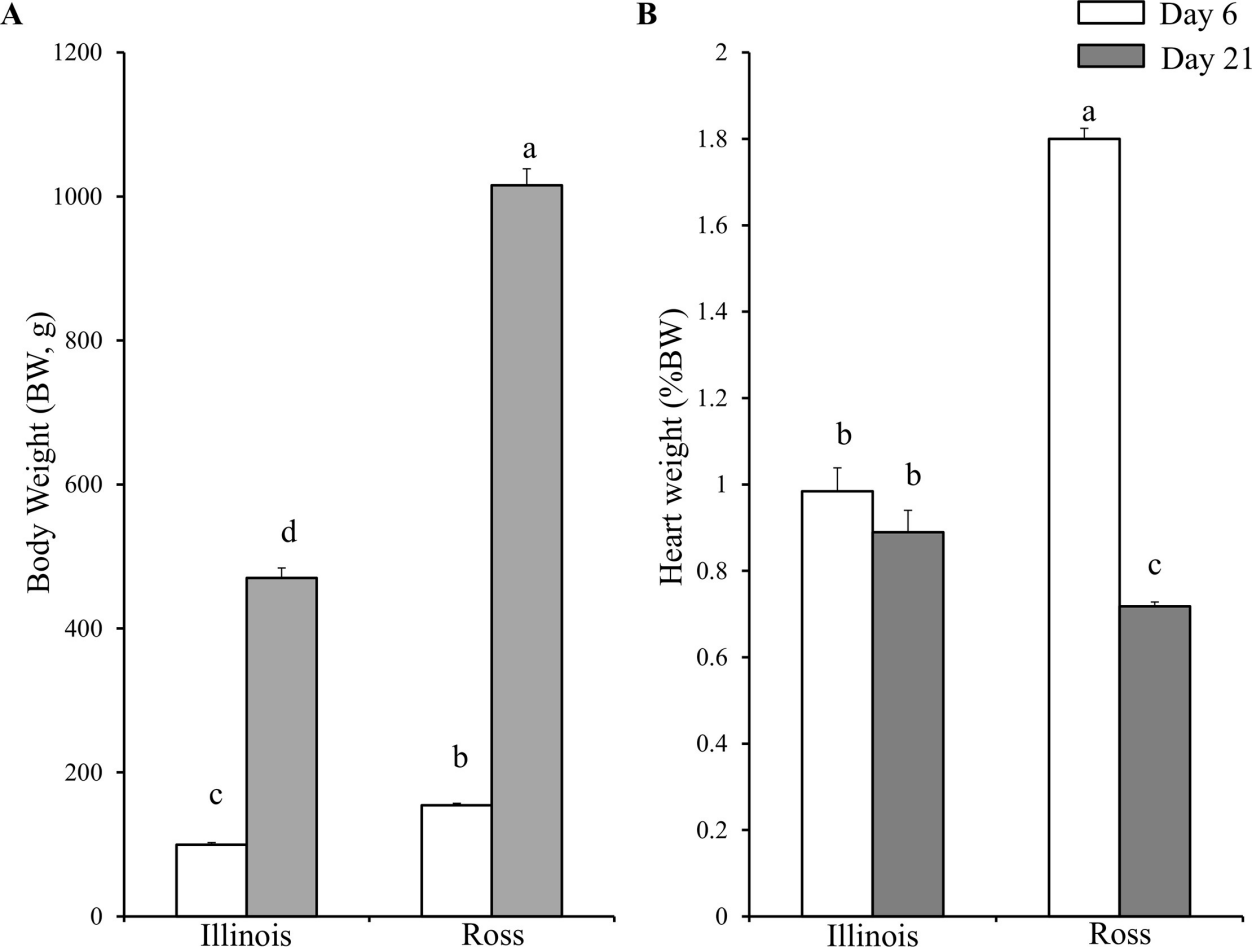
Ross broilers showed faster body growth but relatively slower cardiac development than Illinois broilers. (A) Body weight of two broiler lines at day 6 and 21. (B) Normalized heart weight of two broiler lines at day 6 and 21. Each bar represents Mean±SEM. Different letters (a-d) represent significant differences among different groups.

The body weight (BW) of Ross broilers at 6 days posthatch was about 1.5 times higher (P<0.05) than that of Illinois broilers of the same age. With a faster growth rate, BW of Ross broiler had increased more than 6 times (P<0.0001) by day 21, while BW of Illinois broilers only increased less than 5 times (P<0.0001) by day 21, making its BW less than half of Ross broilers (P<0.0001) (Fig 1A). In contrast to the significant increase of BW, the increase of heart weight was much slower in both broiler lines, making the normalized heart weight decreased. However, only the Ross broiler showed a significant decrease (P<0.0001) of the normalized heart weight. The normalized heart weight of Ross broilers was over 1.5 times (P<0.05) than that of Illinois broilers at day 6, but by day21, it had become smaller (P<0.05) than that of Illinois broilers (Fig 1B).

### RNA-seq output summary and principal component analysis summary

**Fig 2:**
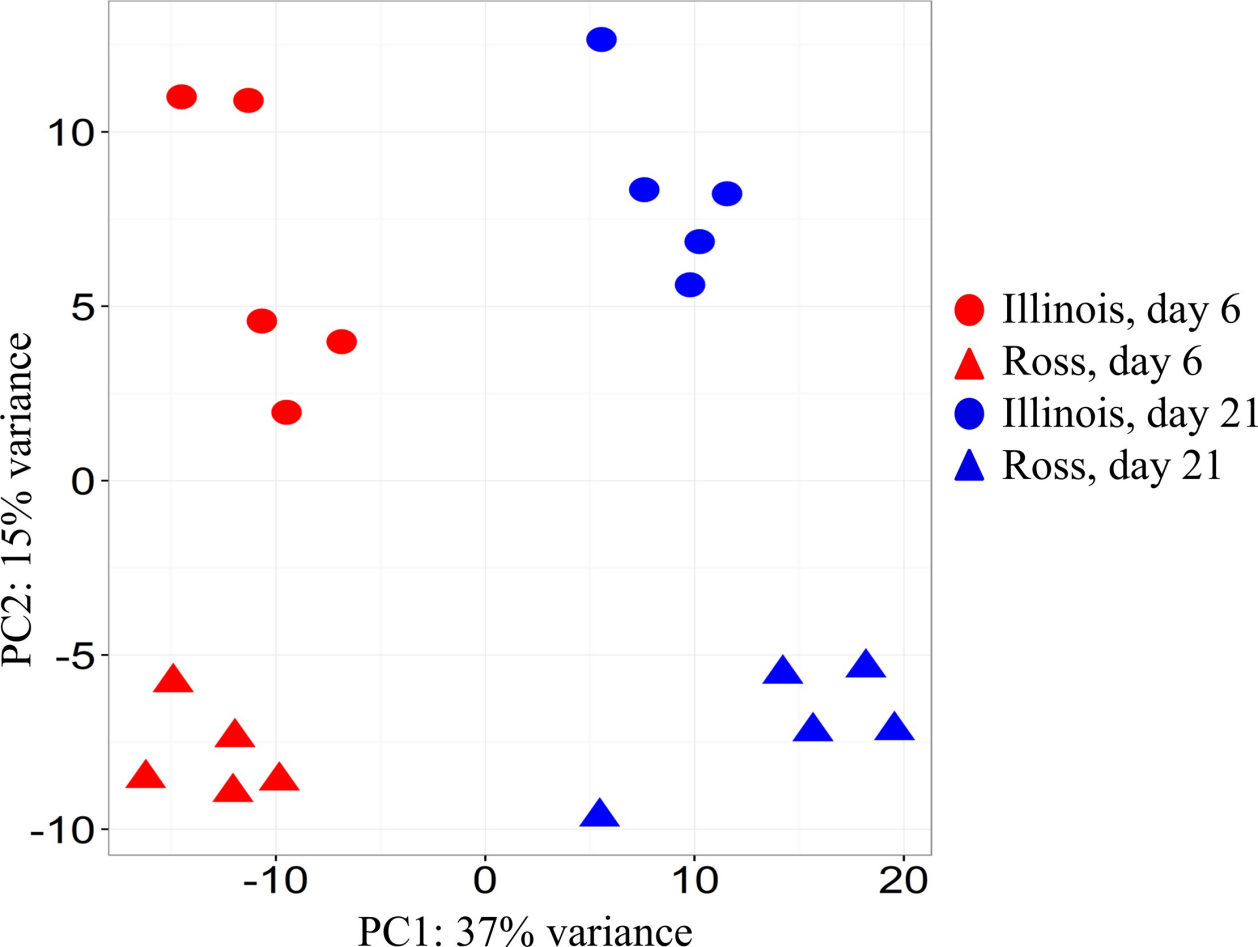
Principal component analysis showed distinct clustering of samples in different groups in different colors. Principal component 1 (PC1) in horizontal axis and PC2 in vertical axis explain 37% and 15% of variation in variance-stabilized normalized counts, respectively.

There were 17 to 32 million 50bp single-end raw reads left for each sample after trimming and filtering. Among these reads, 95–97% of them were mapped to the *Gallus gallus* Galgal5.0 reference genome in the Ensembl database (S2 Table). These reads were mapped to 16,392–18,691 genes in each individual, accounting for 65–75% of the 24,881 annotated genes. With the threshold for read counts for each gene being above 1 count per million in at least five samples, 12,661 genes were retained for differential expression analysis. The principle component analysis (PCA) plot showed clear separation of samples between different lines and different ages. The samples at day 6 and day 21 clustered along principle component 1 (PC1) which explains 37% of variance. Clustering of samples between Ross and Illinois was also distinct along PC2, which explains 15% of variance (Fig 2).

### Gene expression differed between broiler lines and changed as the chicken grew

**Fig 3:**
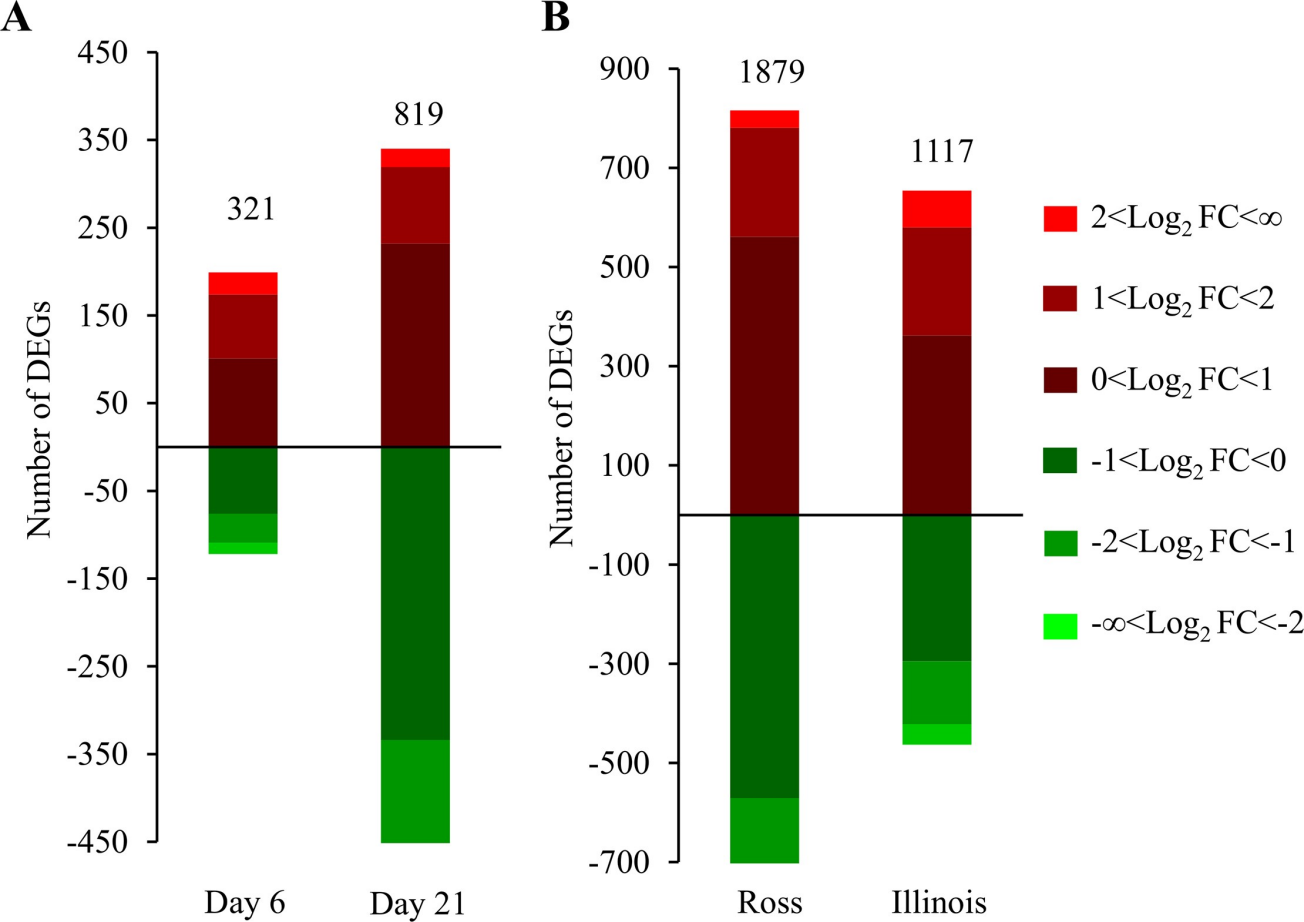
Number of differentially expressed genes (DEGs) with false discovery rate (FDR) <0.05 in different contrasts. (A) Nubmer of DEGs between two broiler lines at day 6 and day 21. (B) Number of DEGs between between day 21 and day 6 within each line. Upregulated and downregulated DEGs are presented in red and blue color. DE genes within different range of Log2 fold change were represented in different intensity of the color.

With FDR below 0.05, 321 genes were significantly differentially expressed between Ross and Illinois at day 6, and this number increased to 819 at day 21(Fig 3A). Only 88 DEGs shared between the two days (S1 File). Among the unique DEGs in each contrast, myosin heavy chain 1E (*MYH1E*) is the top annotated DEG with lower expression (LFC<-3), and guanylate cyclase activator 2A (*GUCA2A*), apolipoprotein A4 (*APOA4*) and B (*APOB*) are the top annotated DEGs with higher expression (LFC>3) in Ross than in Illinois broilers at day 6 (Table 1). Five genes [fatty acid binding protein 4 (*FABP4*), WD repeat domain 17 (*WDR17*), nuclear receptor subfamily 4 group A member 3 (*NR4A3*), hemoglobin subunit beta subunit A (*HBBA*) and retinol binding protein 7 (*RBP7*)] showed much higher expression (LFC>3) in Ross than Illinois broilers at day 21 (Table 1).

**Table 1:** Top unique DEGs with |Log_2_(Fold Change)|>3 for each comparison.

Contrast	Gene ID	Gene Name	Log_2_(Fold Change)
Ross vs. Illinois at day 6	ENSGALG00000029606	*MYH1E*	-4.48571
	ENSGALG00000027269	*GUCA2A*	5.199285
	ENSGALG00000007109	*APOA4*	4.385074
	ENSGALG00000016491	*APOB*	3.238129
Ross vs. Illinois at day 21	ENSGALG00000030025	*FABP4*	3.747346
	ENSGALG00000043106	*WDR17*	3.673439
	ENSGALG00000013568	*NR4A3*	3.62707
	ENSGALG00000028273	*HBBA*	3.448613
	ENSGALG00000002637	*RBP7*	3.36089
Day21 vs. Day6 in Ross broilers	ENSGALG00000016491	*APOB*	-5.24655
	ENSGALG00000035219	*ALB*	-5.19727
	ENSGALG00000046217	*PIT54*	-5.08558
	ENSGALG00000004129	*SPP2*	-4.65607
	ENSGALG00000033376	*APOH*	-4.63739
	ENSGALG00000008601	*AHSG*	-4.56889
	ENSGALG00000019845	*GAL9*	-4.51473
	ENSGALG00000007109	*APOA4*	-4.23166
	ENSGALG00000009266	*FGA*	-4.1972
	ENSGALG00000011612	*GC*	-4.11616
	ENSGALG00000015143	*TTR*	-4.04382
	ENSGALG00000023435	*GATM*	-3.9296
	ENSGALG00000030002	*KRT18*	-3.40364
	ENSGALG00000022815	*GAL1*	-3.36562
	ENSGALG00000030025	*FABP4*	3.534243
	ENSGALG00000002637	*RBP7*	3.45057
Day21 vs. Day6 in Illinois broilers	ENSGALG00000029606	*MYH1E*	-4.0246
	ENSGALG00000005226	*MYOZ1*	-3.01199

**Fig 4:**
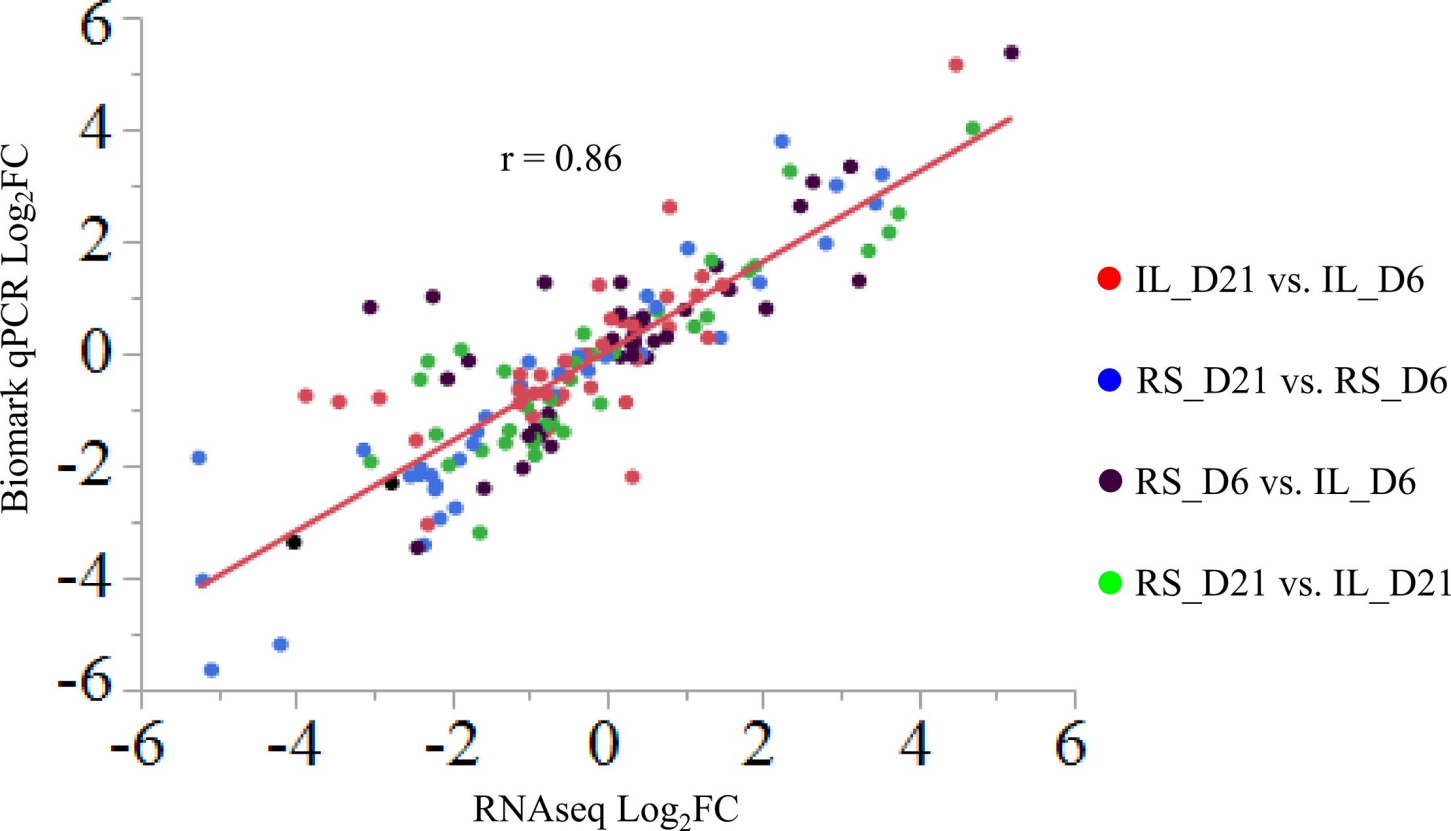
Linear regression fitted between Log2 fold change (FC) in RNA-seq analysis and -ΔΔCt in Biomark qPCR assay of 39 selected DEGs. Different contrasts were marked in different colors. Different groups in the comparisons were labeled as Line_Age (RS = Ross; IL = Illinois; D6 = day 6; D21 = day 21). Pearson correlation coefficient is labeled as “r”. Log2FC in Biomark assay equals -ΔΔCt for each comparison. Average Ct value for each group is the mean of samples in that group. Geometric mean expression of three housekeeping genes including *GAPDH*, *H6PD* and *RPS13* was used for normalization of Ct values.

For the contrast of day 21 vs. day 6 within the same line, more DEGs (n = 1879) were found in Ross than Illinois broilers (n = 1117) (Fig 3B). Among those DEGs, 508 genes were shared between the two lines, of which most genes were regulated similarly except for tubulin beta 6 (*TUBB6*) and *GUCA2A* (S1 File). Expression of both genes was downregulated in Ross but upregulated in Illinois broilers from 6 to 21 days of age (S1 File). Among the annotated unique DEGs in day 21 vs. day 6 contrast in Ross broilers, expression of 14 genes including *APOB*, albumin (*ALB*), *PIT54*, secreted phosphoprotein 2 (*SPP2*), apolipoprotein H(*APOH*) and A4 (*APOA4*), alpha 2-HS glycoprotein (*AHSG*), gallinacin-1 (*GAL1*) and 9 (*GAL9*), fibrinogen alpha chain (*FGA*), gc-globulin (*GC*), transthyretin (*TTR*), glycine amidinotransferase (*GATM*), and Keratin 18 (*KRT18*), showed great reduction (LFC<-3), while expression of *FABP4* and *RBP7* showed large increase (LFC>3) from day 6 to day 21 (Table 1). Among the annotated unique DEGs in the day 21 vs. day 6 contrast in Illinois broilers, expression of *MYH1E* and myozenin 1 (*MYOZ1*) showed great reduction (LFC<-3) from day 21 to day 6 (Table 1), whereas the DEGs with large increase of expression (LFC>3) are all among the shared DEGs with Ross broiler. We validated the RNAseq analysis results by using the Fluidigm Biomark qPCR to assay expression of 39 DEGs that had large and small fold changes in RNAseq. The two detection techniques yielded a high correlation at 0.86 between Log2FC in RNAseq and qPCR (Fig 4).

### Top pathways in each comparison predicted by IPA

**Fig 5:**
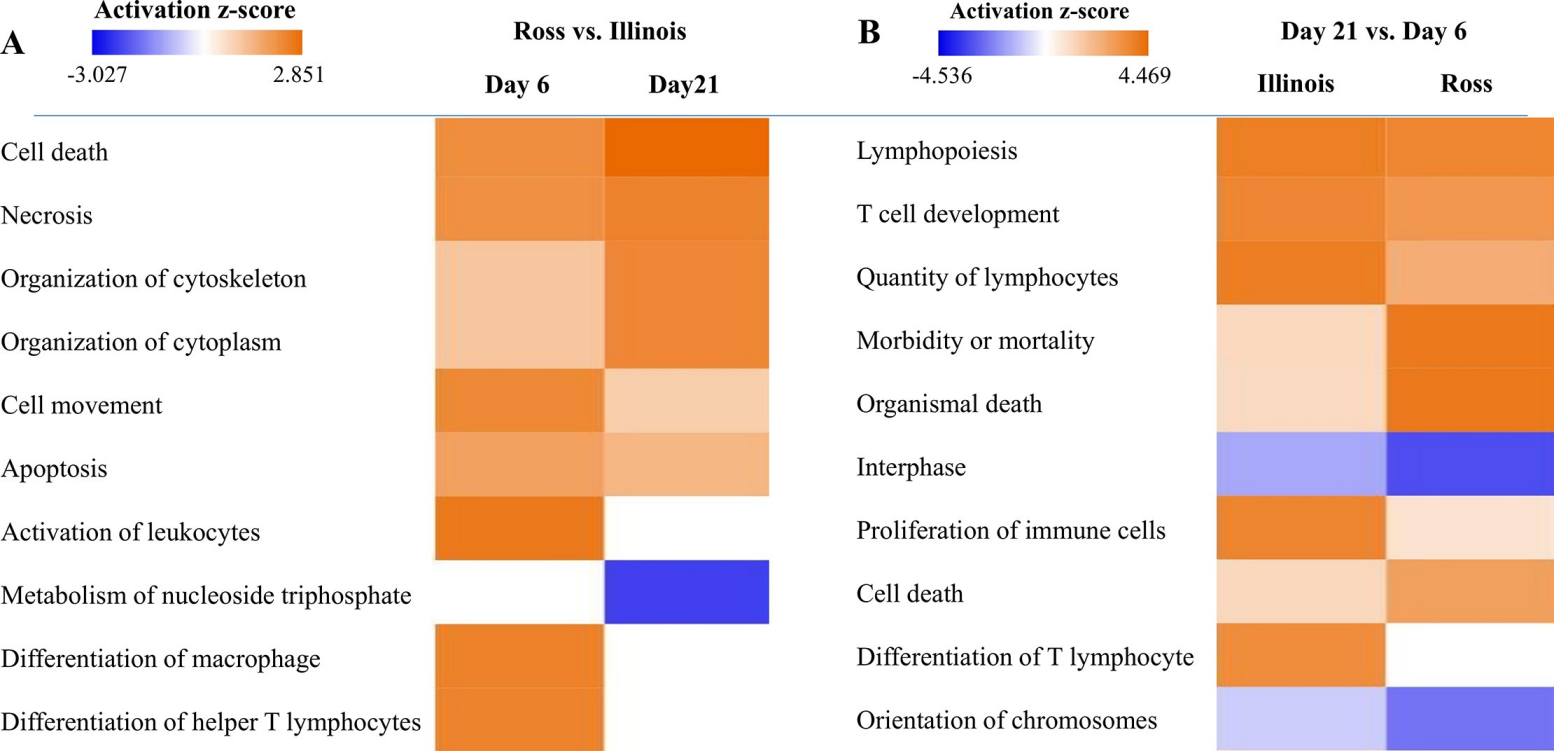
Increased cell apoptosis in Ross broilers and active immune cell development was predicted by IPA. (A) Top 10 Diseases and Biofunctions predicted for Ross vs. Illinois at day 6 and day 21. (B) Top 10 Diseases and Biofunctions predicted for Day 21 vs. Day 6 in the two lines. Prediction of activation or inhibition of a disease or biofunction is calculated as negative or positive z-score and colored in blue or orange, respectively, in the heat map. The intensity of the color in the heat map based on |z-score| indicates robustness of the prediction. The range of activation z-score in each heat map is shown as a bar with minimum and maximun at two ends.

With the DEGs in each comparison as input in Ingenuity Pathway Analysis (IPA), we identified the pathways that were significantly changed from day 6 to 21 in the Ross and Illinois broilers and the pathways that were differentially regulated between the two lines at day 6 and at day 21. Table 2 lists the top five pathways for each contrast. At day 6, all of the top five pathways are involved in immune regulation, with all major genes showing higher expression in Ross than Illinois, indicating that immune cell development may be more rapid in Ross broilers. Among the DEGs that contribute to prediction of these pathways, signal transducer and activator of transcription 1 (*STAT1*), MHC class I antigen (*BF2*), T-cell surface glycoprotein (*CD4*) and Phosphoinositide-3-Kinase Regulatory Subunit 5 (*PIK3R5*) are key genes involved in multiple pathways. At day 21, expression of several genes controlling cell proliferation in the heart of Ross broilers are decreased relative to Illinois birds, including cyclin dependent kinases (CDKs), minichromosome maintenance complex components (MCMs), proliferating cell nuclear antigen (PCNA), origin recognition complex subunit 5 (ORC5), and replication protein A2 (RPA2) in the “Cell Cycle Control of Chromosomal Replication” pathway. Additionally, the lower expression of genes encoding ATP synthase proteins (ATP5s), ubiquinol-cytochrome C reductase core proteins (UQCRs), heat shock proteins (HSPs) and proteasome subunit (PSMs) in Ross broilers also suggest lower mitochondrial respiratory activity, unfolded protein response and protein ubiquitination activity. These top pathways also correspond to the top diseases and biofunctions predicted by IPA (Fig 5). As shown in Fig 5A, higher activity of leukocyte activation, macrophage differentiation and T cell differentiation was predicted in Ross broilers than Illinois broilers at day 6. In addition, higher activity of cell apoptosis was predicted in Ross than Illinois broilers at both days.

**Table 2:** Top significant pathways (p<0.05) predicted by IPA for contrasts between lines or ages.

Contrasts	Pathways	Important DEGs contributing to prediction	Ratios
Ross vs. Illinois at day 6	IL-12 Signaling and Production in Macrophages	***APO(A4/B)*, *IKBKE*, *IRF(1/8)*, *JMJD6*, *PIK3R5*, *STAT1*, *SPI1***	9/146
	Th1 and Th2 Activation Pathway	***BF2*, *CD4*, *IL18R1*, *IRF1*, *PIK3R5*, *SOCS1*, *SPI1*,*STAT1***	10/185
	Antigen Presentation Pathway	***B2M*, *BF2*, *NLRC5*, *TAP(1/2)***	5/38
	Th1 Pathway	***BF2*, *CD4*, *IL18R1*,*IRF1*, *PIK3R5*, *SOCS1*, *STAT1***	8/135
	Production of Nitric Oxide and Reactive Oxygen Species in Macrophages	***APO(A4/B)*, *IKBKE*, *STAT1*, *PIK3R5*, *IRF(1/8)*, *RHOB*, *SPI1***	9/194
Ross vs. Illinois at day 21	Unfolded Protein Response	*ATF4*, ***BCL2*,** *HSP (90B1/A2/A5/A8/H1)*, *PDIA6*, *SREBF1*, *INSIG*	12/55
	Oxidative Phosphorylation	*ATP5(F1A/MC1/MC3/MF/MG/PD/PF)*, *UQCR(C1/FS1/H/Q)*	16/109
	Cell Cycle Control of Chromosomal Replication	*CDK(1/2/7)*, *MCM(2/3/5/6)*, *ORC5*, *PCNA*, *RPA2*	10/56
	Mitochondrial Dysfunction	*ATP5(F1A/MC1/MC3/MF/MG/PD/PF)*, *UQCR(C1/FS1/H/Q)*	17/171
	Protein Ubiquitination Pathway	*DNAJ(A1/B9/ /C15)*, *HSP(A2/A5/A8/B7/H1)*, *PSM(A3/A7/B3/D3)*	20/265
Day 21 vs. Day 6 in Ross	Role of BRCA1 in DNA Damage Response	*BRCA(1/2)*, *BARD1*, *FANC(A/B/C/D2/F/L)*, *PLK1*, *RAD51*	28/80
	Superpathway of Cholesterol Biosynthesis	*ACAT2*, *DHCR(7/24)*, *IDI1*, *LBR*, *LSS*, *NSDHL*,*SC5D*, *SQLE*	17/28
	Cell Cycle Control of Chromosomal Replication	*CDK(1/2)*, *DNA2*, *MCM(2/3/4/5/6/8)*, *ORC(1/4/5)*, *RPA(1/2/3)*	23/56
	Mitotic Roles of Polo-Like Kinase	*KIF(11/23)*, *CCNB2*, *CDC(2/7/20/25A/ 27)*, *PLK(1/4)*, *PRC1*	20/66
	Role of CHK Proteins in Cell Cycle Checkpoint Control	*ATR*, *BRCA1*, *CDC25A*, *CDK(1/2)*, *CHEK(1/2)*, *CLSPN*, *EIF(1/7/8)*, *PCNA*, *PLK1*, *RFC(2/3/4)*, *RPA1*	18/57
Day 21 vs. Day 6 in Illinois	EIF2 Signaling	***ATF4*, *EIF3G*, *PIK3R5*, *RPL(3/7/8/9/11/13/21/23/24/29/30/31/32)***	43/221
	Th1 and Th2 Activation Pathway	***BF2*, *BLB1*, *CD(247/3D/3E/4)*, *MHCDM(A/B2)*, *STAT(3/4/5B)***	24/185
	Superpathway of Cholesterol Biosynthesis	*ACAT2*, *DHCR(7/24)*, *IDI1*, *LBR*, *LSS*, *NSDHL*, *SC5D*, *SQLE*	10/28
	Antigen Presentation Pathway	***B2M*, *BF2*, *BLB1*,*CD74*, *CIITA*, *MHCDM(A/B2)*, *TAP(1/2/BP)***	11/38
	T Cell Receptor Signaling	***CARD11*, *CD(247/3D/3E/4)*, *IKBKE*, *LCK*, *PIK3R5*, *TXK*, *ZAP70***	16/109

Bold italic and italic font, respectively, indicates higher and lower expression of differentially expressed genes (DEGs) in Ross compared to Illinois or at day 21 compared to day 6. Genes within the same family or complex are labeled with the member or subunit names in the brackets. Ratios = (Number of DEGs in a pathways) / (Total number of genes in the pathway).

Contrasts between the two times showed significant changes as the broilers grew from day 6 to 21. Ross broilers had lower cell proliferation activity as shown by decreased expression of DEGs involved in several cell cycle related pathways including “Role of BRCA1 in DNA Damage Response”, “Cell Cycle Control of Chromosomal Replication”, “Mitotic Roles of Polo-Like Kinase” and “Role of CHK Proteins in Cell Cycle Checkpoint Control*”* (Table 2). In Illinois broilers, immune cell maturation or inflammatory response seems to be a major change as indicated by increased expression of DEGs participating “EIF2 signaling”, “Th1 and Th2 Activation Pathway”, “Antigen Presentation Pathway” and “T Cell Receptor Signaling” pathways. Immune cell development and cell apoptosis were enhanced in both lines over time as indicated by the predicted diseases and biofunctions (Fig 5B). However, immune cell development was enhanced much more in Illinois broilers as indicated by the higher z-scores for lymphopoiesis, quantity of lymphocytes, proliferation of immune cells, and differentiation of T lymphocytes. In contrast, apoptosis is enhanced much more in Ross broilers as suggested by the higher z-score for cell death and lower z-scores for interphase and orientation of chromosomes (Fig 5B). Therefore, IPA also predicted higher gene expression activity contributing to morbidity or mortality, and organismal death in Ross broilers (Fig 5B). Both chicken lines showed downregulation of “Superpathway of Cholesterol Biosynthesis” as suggested by reduced expression of genes encoding the important enzymes including acetyl-CoA acetyltransferase 2 (ACAT2), dehydrocholesterol reductases (DHCRs), 3-hydroxy-3-methylglutaryl-CoA reductase (HMCGR) and synthase (HMGCS2), lanosterol synthase (LSS), NAD(P) dependent steroid dehydrogenase-like (NSDHL), sterol-C5-desaturase (SC5D) and squalene epoxidase (SQLE) (Table 2).

## Discussion

Production of modern broilers has been compromised by multiple morbidities such as sudden death, ascites syndrome, hypoxemia, lameness due to inadequate development of other organs including heart [6,16,17], liver [6], intestine [6,17], lungs [16,17], and skeleton ossification [14]. Among these morbidities, sudden death and ascites syndrome due to cardiovascular dysfunction are the major diseases in modern fast-growing broilers. In a study comparing the 1957 ACRBC and the 1991 AA broilers, higher mortality was observed in AA broilers with the most mortality occurring after 21 days due to sudden death syndrome and ascites [8]. Although these diseases occur mainly after 21 days posthatch, their initiation at gene expression and molecular level may begin at earlier ages [10]. Therefore, although there was no incidence of mortality and observable cardiac morbidity in the Ross and Illinois broiler lines during this study, comparison of gene expression in left ventricle tissue between the two broiler lines showed DEGs and differentially regulated pathways at early ages which might explain the different frequency of cardiac dysfunction between Ross and Illinois lines at the later age.

Among the four contrasts between the two lines within time or two times within line, several genes were consistently ranked as top DEGs in multiple contrasts, suggesting their special importance to heart development in the broilers. For example, three apolipoprotein genes *APOA4*, *APOB* and *APOH* which encode proteins that bind and transport lipids in circulatory system and impede cardiac triglyceride accumulation [18] showed distinctly higher expression in Ross than in Illinois at day 6, and also significantly reduced expression from day 6 to day 21 in Ross broilers. In contrast, *FABP4* [19] and *RBP7* [20], which encode lipid binding proteins involved in cellular lipid uptake showed distinctly higher expression in Ross than in Illinois at day 21, and also significantly increased expression from day 6 to day 21 in Ross broilers. Therefore, the decreased expression *APOA4* and *APOB* and increased expression of *FABP4* and *RBP7* in Ross broiler at day 21 may indicate initiation of higher lipid deposition in Ross compared to Illinois broilers. This speculation is supported by the hypothyroid state associated with decreased metabolism maintenance and increased fat deposition in the broilers with ascites [10] and higher carcass fat in AA than ACRBC broilers at the same age when fed the same diet [7]. Excessive lipid accumulation in nonadipocyte cells may lead to their dysfunction and cause lipotoxicity [21]. Studies in New Zealand White rabbits have demonstrated that obese rabbits fed with a high fat diet developed cardiac hypertrophy [22], showed increased heart rate [23] and cardiac output [24], reduced left ventricular contractility [25] and diastolic compliance [26] with two-fold increase of fat weight in both ventricles. Zucker diabetic fatty rats which exhibited elevated myocardial triglycerides also showed poor systolic function [27]. In leptin-deficient *ob/ob* mice, marked accumulation of lipid droplets within cardiac myocytes is paralleled by cardiac diastolic dysfunction [28]. In addition, excessive lipid in myocardium has also been correlated with systolic dysfunction in humans [29]. In terms of gene expression, transgenic expression of APOB in heart reduces lipotoxic cardiomyopathy in mice [30], but overexpression of *FABP4* in cardiomyocytes aggravates cardiac hypertrophy in mice under pressure overload through activation of extracellular signal-regulated kinase (ERK) signaling [31]. In humans, *FABP4* has been identified as a biomarker for cardiac metabolism and physiopathology including left ventricular hypertrophy and both systolic and diastolic cardiac dysfunction [32].

Interestingly, the postulation that accumulation of myolipids could lead to cell apoptosis in heart [33] is in agreement with the prediction of enhanced cell death, reduced metabolism of nucleoside triphosphate (Fig 5A) and downregulation of cell cycle related pathways (Table 2) in Ross broiler at day 21 by IPA. Despite the increased cell death and reduced cell cycle in both broiler lines from day 6 to day 21, the Ross broiler seemed to show many more changes as predicted by IPA (Fig 5B). Among the cell cycle regulatory pathways with significant change in Ross broilers, “Mitotic Roles of Polo-Like Kinase”, “Cell Cycle Control of Chromosomal Replication”, and “Role of CHK Proteins in Cell Cycle Checkpoint Control” are all top ranked pathways (P<0.01) downregulated in Ross broilers that were treated by mild heat stress at day 42 [9]. With downregulation of the same DEGs such as polo like kinase 1 (PLK1) and PLK4, cyclin B2 (CCNB2), cell-division cycle protein 2 (CDC2), CDC7, CDC20, protein regulator of cytokinesis 1 (PRC1), kinesin family member 11 (KIF11) and KIF23, the “Mitotic Roles of Polo-Like Kinase” pathway has been suggested to be the primary pathway related to hyperthermia-induced apoptosis and thus reduced heart weight in Ross broilers under heat stress. “Cell Cycle Control of Chromosomal Replication” pathway is also one of the top pathways with lower activity in Ross broilers compared to Illinois broilers at day 21. Therefore, the reduced cell proliferation and inhibition of these cell-cycle regulatory pathways may be the reason of decreased normalized heart weight in Ross broilers from day 6 to day 21. This is in agreement with our previous finding that increased apoptosis is the reason of reduced heart in Ross broilers under heat stress. Also, the degeneration of proteins, reduced fractional shortening, and thinner ventricular wall observed in broilers with left ventricular depression [5] support our speculation of higher apoptosis activity in heart of Ross broilers than Illinois broilers.

In addition to increased lipid accumulation, other processes contributing to increased cell apoptosis and cardiac dysfunction may be active in Ross broilers, such as increased mitochondrial dysfunction [34]. Genes encoding subunits of ATP synthase (ATP5s), which catalyzes ATP synthesis, and those encoding subunits of ubiquinol-cytochrome C reductase (UQCRs), which is part of mitochondrial respiratory chain, all showed lower expression in Ross than in Illinois at day 21. These DEGs may contribute to mitochondrial dysfunction and decreased oxidative phosphorylation in Ross broilers (Table 2). This hypothesis is consistent with the increased free radicals and lipid peroxidation [35] as well as enlarged and swollen mitochondria with poorly defined matrices and cristae [36] observed in broilers with ascites. In addition, impaired cardiac mitochondrial oxidative phosphorylation and enhanced mitochondrial oxidative stress also occurred in domestic cats with hypertrophic cardiomyopathy [37], which supports the concept that a similar change may contribute to susceptibility of Ross broilers to cardiac diseases. Oxidative stress could disrupt protein folding mechanisms, leading to increased production of misfolded proteins [38]. However, the lower activity of “Unfolded Protein Response” and “Protein Ubiquitination” pathways (Table 2) in Ross broilers at day 21 may reduce their ability to respond to these misfolded proteins, leading to endoplasmic reticulum stress-induced cell apoptosis [39]. Active expression in cardiomyocytes of heat shock protein family A member 5 (*HSPA5*), which is a master regulator of unfolded protein response, plays a critical role in cardiac development [40] and protection against oxidative stress and ischemia/reperfusion injury [41]. Inhibition of the ubiquitin-proteasome system has also been associated with cardiotoxicity in human, whereas enhancing proteasome activity could improve the outcome of cardiomyopathies and infracted hearts caused by oxidative stress [42]. Therefore, lower expression of *HPSA5*, and lower unfolded protein response and protein ubiquitination activities in Ross than in Illinois broilers at day 21 (Table 2) may be detrimental for cardiac development of these birds.

In contrast to expression changes of lipid-related genes from day 6 to 21 in Ross broilers, the top-ranked unique DEGs in Illinois broilers with highest fold change from day 6 to 21 are involved in muscle function and development, such as *MYH1E* and *MYOZ1*. *MYH1E* is a orthologous gene of myosin heavy chain 4 (*MYH4*) in mammals which is predominantly expressed in fast-twitch muscle fiber [43]. *MYOZ1* which encodes calsarin-2 is also expressed mainly in fast-twitch skeletal muscle fiber and at lower level in cardiac tissue [44]. Previous studies on these genes are mostly focused on their expression and functions in skeletal muscle rather than cardiac muscle. However, expression of *MYOZ1* in left and right atria has been reported as a cause of a type of arrhythmia—atrial fibrillation [45]. These findings indicate that *MYH1E* and *MYOZ1* may have important functions in the heart, worthy of future investigation.

Enhanced activity of immune cell development, proliferation and differentiation appears to be a common change in both broiler lines from day 6 to day 21 as predicted by IPA (Fig 5B). However, Ross broilers seem to have less change than Illinois broilers (Fig 5B), possibly due to tradeoff between fast growth and immune cell development, which is common among modern commercial broilers [46, 47]. Four among the five top pathways for day 21 vs. day 6 contrast in Illinois broilers are involved in immune system (Table 2). “EIF2 signaling” pathway regulates proinflammatory cytokine expression and its up regulation has been related to enhanced resistance to NDV in chickens [48]. “Antigen Presentation Pathway” in antigen presenting cells and “Th1 and Th2 Activation Pathway” regulates T cell differentiation into T helper cells, and “T Cell Receptor Signaling” pathway is also important for T cell maturation, homeostasis and activation [49]. Despite the greatly enhanced immune response in Illinois broilers from day 6 to 21, the immune cell activity appears to be higher at day 6 in Ross than in Illinois broilers, because the top five pathways for the contrast were involved in immune system regulation, and they all showed higher activity in Ross with higher expression of major genes (Table 2). The high activity of immune-regulating pathways in normal heart tissue is unexpected, since the heart is composed primarily of muscle cells. However, there are also resident immune cells such as macrophages and dendritic cells in the myocardium. These cells play important roles to phagocytose and store the undigested cholesterol and prevent cardiac inflammation [50]. In addition, macrophages that abound in atrioventricular nodes in human and mouse have been reported to assist normal electrical conduction [51]. IL-12 production in macrophages protects against myocarditis by increasing macrophage and neutrophil populations in heart [52], which corresponds to the top rank of “IL-12 Signaling and Production in Macrophages” pathway in Ross vs. Illinois contrast at day 6 (Table 2). Therefore, development of immune cells in chicken hearts as they grow may be also necessary for normal cardiac function. To our knowledge, ours is the first report of significant transcription differences in immune-system genes and pathways potentially related to cardiac function in broiler chicken. This highlights the need for future research to further characterize the role of the immune system in normal and dysfunctional cardiac tissue in chickens.

In summary, we identified genes that may be associated with cardiac development from day 6 to day 21 in Ross and Illinois broilers, and differences between the lines. From pathway analysis with the DEGs, we infer that immune cell development and function are dynamic in cardiac tissue from day 6 to 21 during posthatch development of chickens. In addition, we speculate that the differential gene expression and altered pathways observed in Ross broilers may indicate initiation or progression of decreased cell proliferation combined with increased cell apoptosis due to increased lipid accumulation, oxidative stress and endoplasmic reticulum stress may contribute to the slowed cardiac development in the these birds. These hypotheses, although still need to be validated by histological and cellular studies, are consistent with the previous reports about reduced relative heart size [6], degradation of cardiac muscle protein [5], hypothyroid state [10], higher fat percentage [7], altered mitochondria [37], and increased free radical [36] in modern fast-growing broilers. These findings lead to a better understanding of chicken cardiac development and genetic regulation underpinning the compromised cardiac health in modern fast-growing broilers, and also provide direction for future studies at the cellular level and some potential targets for future breeding for improved cardiac health in modern broilers.

## Supporting information

S1 Table Primers used in Fluidigm Biomark q-PCR for the validations of RNA-seq data. (DOCX)

S2 Table Statistical summary of sequence reading, mapping and counting in RNAseq analysis. (DOCX)

S1 File Lists of shared DEGs between contrasts with their Log2(Fold change), p-values and false discovery rate. Sheet 1 contains shared DEGs of Ross vs. Illinois contrasts between day 6 and day 21, and Sheet 2 contains shared DEGs of day21 vs. day6 contrasts between Ross and Illinois broiler lines.
(XLSX)
