# Supplementary material for: Distinct genes and pathways associated with transcriptome differences in early cardiac development between fast- and slow-growing broilers
Source: PLoS One. 2018 Dec 5;13(12):e0207715. doi: 10.1371/journal.pone.0207715 (PMC6281182; doi:10.1371/journal.pone.0207715)
Supplement: S1 Table — (DOCX) [file pone.0207715.s001.docx]

**S1 Table. Primers used in Fluidigm Biomark q-PCR for the validations of RNA-seq data.**

| **Gene Symbol** | **GenBank Accession Number** | **Forward Primer**  **5’-3’** | **Reverse Primer**  **5’-3’** |
| --- | --- | --- | --- |
| *ALB* | [NM_205261](https://www.ncbi.nlm.nih.gov/nuccore/NM_205261.2) | ACCAGGACAACAGAGTGTCA | GATTGCAGGGGCATACAAGAA |
| *APOB* | [NM_001044633](https://www.ncbi.nlm.nih.gov/nuccore/NM_001044633.1) | CCCATGGGAACACAGTTTCAA | TGGCACCTGAATTCCACTCTA |
| *AVD* | NM_205320 | TTCACAGGCACCTACATCACA | GGTGTTTTGTGTCCCATGCA |
| *BMP10* | [NM_001278046](https://www.ncbi.nlm.nih.gov/nuccore/NM_001278046.1) | GGACATCTATGCCATCTGCAAA | CGAACTCCTATGACGCCAAC |
| *BRCA1* | [NM_204169](https://www.ncbi.nlm.nih.gov/nuccore/NM_204169.1) | TGCTGCTGTGGACCTTTCA | TTCACTACAGAGGCACCACAAA |
| *CCNB2* | [NM_001004369](https://www.ncbi.nlm.nih.gov/nuccore/NM_001004369.1) | CTCCAGCTGCTGTGAATCAA | ACATCCATAGGGACAGGAGAC |
| *CDK1* | [NM_205314](https://www.ncbi.nlm.nih.gov/nuccore/NM_205314.1) | CCACAGCCATGGAGGATTACA | TGTGGCGCCCTTTATACACA |
| *CMPK2* | XM_015284945 | AGGCTGAACTGGAAGCTAACA | CTTGGCACGCAGGATTCAC |
| *CXCR4* | [NM_204617](https://www.ncbi.nlm.nih.gov/nuccore/NM_204617.2) | TATGGACGGCAGCATGGA | AATCTCCTCCGAGCCATTGT |
| *FABP4* | NM_204290 | AAAAGTGGTGGATGGGAACC | GCTTCCTCATGCTCTTTCGT |
| *FAM196B* | [XM_004944784](https://www.ncbi.nlm.nih.gov/nuccore/XM_004944784.2) | ACATTTCCTGTGGGTAAACTCA | GCATCAGAAGAGTGGTCCAA |
| *FGA* | XM_015284945 | GCTCTGCAGAACAGCATCCA | GCTCCCTTTGCAAGCTCGTA |
| *FOXM1* | [NM_001012955](https://www.ncbi.nlm.nih.gov/nuccore/NM_001012955.1) | GTGAAGCAAGGCATGGAGAA | GGTATGGAAACAGCAGCAGAA |
| *GADL1* | [XM_004939424](https://www.ncbi.nlm.nih.gov/nuccore/XM_004939424.2) | AAGCATGACCTCTGGCTTCA | GAAGAAGTCTGCGATGCTTCC |
| *GAPDH* | NM_204305 | GTGCTGGCATTGCACTGAA | CACAACACGGTTGCTGTATCC |
| *GUCA2A* | NM_001197038 | CTCTCCCTGAGGAATTCCAACC | ATCAGCTTCACTGGCAACCA |
| *H6PD* | [XM_425746](https://www.ncbi.nlm.nih.gov/nuccore/XM_425746.5) | ATGTACCGGGTGGACCACTA | AACTGACGGTTCTGATCTCGAAA |
| *HSPA5* | [NM_205491](https://www.ncbi.nlm.nih.gov/nuccore/NM_205491.1) | TTTCTGCCATGGTCCTGACAA | AGGCTGGCACAGTAACAACA |
| *HSPA8* | [NM_205003](https://www.ncbi.nlm.nih.gov/nuccore/NM_205003.2) | CTGGCAAGGAGAACAAGATCAC | TTCCTGAACCATCCGCTCAA |
| *HAP90AA1* | [NM_001109785](https://www.ncbi.nlm.nih.gov/nuccore/NM_001109785.1) | ACACATGCCAACCGCATTTA | CCTCCTCAGCAGCAGTATCA |
| *LCP2* | [NM_204701](https://www.ncbi.nlm.nih.gov/nuccore/NM_204701.1) | TCCTCTACCAGGCAACAATGAC | GGGGTTTCGTGCTTCTGTCTA |
| *MHCIY* | [NM_001184719](https://www.ncbi.nlm.nih.gov/nuccore/NM_001184719.1) | GCCGGAACGCTACAACAAA | TCCAGGATGTCACAGCCAAA |
| *MSTN* | NM_001001461 | CGAGATCTTGCTGTCACATTCC | ACCGTTTCGGTGTGTCTGTA |
| *MYBL1* | NM_204848 | ATGGAACACTTGCACACTCA | TCTGGTGAGGCATACTGGTA |
| *MYBPC1* | [XM_015287663](https://www.ncbi.nlm.nih.gov/nuccore/XM_015287663.1) | GTTGAAGCTAGTGACCGTGTA | TCAACAGCAGCACTTGCTA |
| *MYBPH* | NM_001031028 | ACGTGGTGGAGATCTGCAAA | CTTGTAGCGGGTGGAGAGAAA |
| *MYH7* | [NM_001001302](https://www.ncbi.nlm.nih.gov/nuccore/NM_001001302.1) | ATTTCTGCCCAGCTCCAGAA | TGCCTCTAGTTCCTCCTCCA |
| *MYL1* | [NM_001044632](https://www.ncbi.nlm.nih.gov/nuccore/NM_001044632.1) | TCAACAAGATCCTGGGCAACC | GCATGGGCAGGAACTCTTCA |
| *NR4A3* | XM_015282405 | CCGCTTTCAGAAGTGTCTCA | CCGACCTCTTCTCCCTTTCA |
| *OASL* | NM_205041 | AGCTTCACAGAACTGCAGAA | TCCTTGTACCAGTGCTTGAC |
| *PDK4* | NM_001199909 | TCTCCGCTCTCCATCAAGCA | TCTTGTCGCAGGAACGCAAA |
| *PIT54* | NM_207180 | TGGGGTGAACACAACTGCTA | ACGATGTTTCTGTCTCCTCCA |
| *PLK1* | [NM_001030639](https://www.ncbi.nlm.nih.gov/nuccore/NM_001030639.1) | AGCCAAACCCTCTGAGAGAA | TGCTAACCCAGAAGATGGGAA |
| *PROKR2* | NM_001145229 | TCTACTAACGCTCTCCTGGCTA | CGTGGTTTCAGTGGGTGAAC |
| *RBP7* | [XM_417606](https://www.ncbi.nlm.nih.gov/nuccore/XM_417606.4) | TGGAACCTGGAACCTTGTCA | TCTTGCGTGTTGCAAAGTCA |
| *RPS13* | [NM_001001783](https://www.ncbi.nlm.nih.gov/nuccore/NM_001001783.1) | CTTGCTAAGAAAGGCTTGACTCC | CAAAACGAACCTGGGCAACA |
| *RRM2* | [XM_001231544](https://www.ncbi.nlm.nih.gov/nuccore/XM_001231544.4) | TCTCTGGAAGGCAAGACCAA | GTTGTCTGTGGGCTTTGACA |
| *SIK1* | NM_204682 | GAGCAGCAGAGAGGGAAGAAA | GGAGTAGCTGGTGATGCTGAA |
| *SLN* | NM_001302187 | AGAGCCCCAGAGGCAGAA | GTCCTTGAGCTCAGCCTTCA |
| *SMC2* | [NM_205230](https://www.ncbi.nlm.nih.gov/nuccore/NM_205230.1) | GAAGAGCTCGACCGAAAGAA | GCATCGAGAAGATGGAACCA |
| *SOCS2* | [NM_204540](https://www.ncbi.nlm.nih.gov/nuccore/NM_204540.1) | CGTGCTGATGTGCAAGGAC | ACGTGTACAGGGGTTTGTTCA |
| *TNNT3* | [NM_204922](https://www.ncbi.nlm.nih.gov/nuccore/NM_204922.2) | GCAAGCCCTTGAACATTGAC | ACCAGTCCCACAGTTCCTTA |
| *VAV3* | [NM_206863](https://www.ncbi.nlm.nih.gov/nuccore/NM_206863.3) | ACGTCCTGCAAAGTCTGTCA | CCAGCTCCACACTTAGAGCATA |
