# Supplementary material for: Distinct genes and pathways associated with transcriptome differences in early cardiac development between fast- and slow-growing broilers
Source: PLoS One. 2018 Dec 5;13(12):e0207715. doi: 10.1371/journal.pone.0207715 (PMC6281182; doi:10.1371/journal.pone.0207715)
Supplement: S2 Table — (DOCX) [file pone.0207715.s002.docx]

**S2 Table. Statistical summary of sequence reading, mapping and counting in RNAseq analysis.**

| **Lines** | **Age (DPH)** | **Bird No.** | **Raw Reads** | **Mapped Reads** | **% of Mapped Reads** | **Detected Genes** | **Transcriptome Coverage**  **Coverage** |
| --- | --- | --- | --- | --- | --- | --- | --- |
| Illinois | 6 | 212 | 24,434,717 | 23,652,115 | 96.8% | 16,636 | 66.86% |
| Illinois | 6 | 213 | 26,024,574 | 25,116,198 | 96.5% | 17,138 | 68.88% |
| Illinois | 6 | 218 | 22,357,592 | 21,694,517 | 97.0% | 16,840 | 67.68% |
| Illinois | 6 | 220 | 25,398,180 | 24,588,550 | 96.8% | 17,009 | 68.36% |
| Illinois | 6 | 222 | 17,679,777 | 17,120,780 | 96.8% | 16,392 | 65.88% |
| Illinois | 21 | 231 | 32,094,909 | 31,068,055 | 96.8% | 17,340 | 69.69% |
| Illinois | 21 | 232 | 28,269,677 | 27,148,073 | 96.0% | 16,973 | 68.21% |
| Illinois | 21 | 234 | 29,029,412 | 27,843,020 | 95.9% | 17,423 | 70.02% |
| Illinois | 21 | 235 | 26,046,866 | 25,117,397 | 96.4% | 17,275 | 69.43% |
| Illinois | 21 | 236 | 22,949,521 | 22,027,006 | 96.0% | 16,921 | 68.01% |
| Ross | 6 | 214 | 21,894,108 | 21,244,688 | 97.0% | 16,856 | 67.75% |
| Ross | 6 | 215 | 23,515,811 | 22,405,967 | 95.3% | 18,691 | 75.12% |
| Ross | 6 | 217 | 25,161,547 | 24,370,630 | 96.9% | 17,978 | 72.57% |
| Ross | 6 | 219 | 20,031,731 | 19,346,480 | 96.6% | 17,097 | 68.71% |
| Ross | 6 | 221 | 19,332,426 | 18,648,904 | 96.5% | 17,042 | 68.50% |
| Ross | 21 | 224 | 18,745,134 | 18,094,426 | 96.5% | 16,840 | 67.68% |
| Ross | 21 | 225 | 20,661,503 | 19,960,274 | 96.6% | 17,006 | 68.35% |
| Ross | 21 | 226 | 23,053,338 | 22,283,917 | 96.7% | 17,103 | 68.74% |
| Ross | 21 | 228 | 24,369,644 | 23,524,154 | 96.5% | 16,943 | 68.10% |
| Ross | 21 | 229 | 26,300,278 | 25,436,132 | 96.7%  % | 17,268 | 69.40% |

The percentage of mapped reads was calculated as the number of mapped reads divided by the number of raw reads; Transcriptome coverage was calculated as the number of detected genes (genes with at least one mapped read) divided by the total number of genes in the Galgal5.0 genome, which is 24881.
